# Acute Csk inhibition hinders B cell activation by constraining the PI3 kinase pathway

**DOI:** 10.1073/pnas.2108957118

**Published:** 2021-10-21

**Authors:** Wen Lu, Katarzyna M. Skrzypczynska, Arthur Weiss

**Affiliations:** ^a^Howard Hughes Medical Institute, University of California, San Francisco, CA 94143;; ^b^Rosalind Russell and Ephraim P. Engleman Rheumatology Research Center, Department of Medicine, University of California, San Francisco, CA 94143;; ^c^Department of Microbiology and Immunology, University of California, San Francisco, CA 94143

**Keywords:** B cell receptor signaling, CSK, PI3 kinase, Src-family kinases

## Abstract

T cell antigen receptor (TCR) and B cell antigen receptor (BCR) signaling are initiated and tightly regulated by Src-family kinases (SFKs). SFKs positively regulate TCR signaling in naïve T cells but have both positive and negative regulatory roles in BCR signaling in naïve B cells. The proper regulation of their activities depends on the opposing actions of receptor tyrosine phosphatases CD45 and CD148 and the cytoplasmic tyrosine kinase C-terminal Src kinase Csk. Csk is a major negative regulator of SFKs. Using a PP1-analog-sensitive Csk (Csk^AS^) system, we have previously shown that inhibition of Csk^AS^ increases SFK activity, leading to augmentation of responses to weak TCR stimuli in T cells. However, the effects of Csk inhibition in B cells were not known. In this study, we surprisingly found that inhibition of Csk^AS^ led to marked inhibition of BCR-stimulated cytoplasmic free calcium increase and Erk activation despite increased SFK activation in B cells, contrasting the effects observed in T cells. Further investigation revealed that acute Csk^AS^ inhibition suppressed BCR-mediated phosphatidylinositol 3,4,5-trisphosphate (PIP3) production in B cells. Restoring PIP3 levels in B cells by CD19 cross-linking or SHIP1 deficiency eliminated the negative regulatory effect of Csk^AS^ inhibition. This reveals the critical role of Csk in maintaining an appropriate level of SFK activity and regulating PIP3 amounts as a means of compensating for SFK fluctuations to prevent inappropriate B cell activation. This regulatory mechanism controlling PIP3 amounts may also contribute to B cell anergy and self-tolerance.

## Significance

B lymphocytes recognize pathogenic antigens and become activated via their B cell receptors (BCR). This BCR-dependent activation is controlled by Src-family kinases (SFKs). It is unclear how B cells tolerate the fluctuations of SFK activities and maintain unresponsiveness in the absence of foreign antigens. Using a chemical-genetic system, we acutely inhibited C-terminal Src kinase to enhance the SFK activity in mouse B cells. Surprisingly, we observed marked inhibition of BCR-downstream signaling due to associated impairment of the phosphatidylinositol-trisphosphate pathway. These results reveal the critical importance of maintaining a proper amount of SFK activity in quiescent B cells for appropriate BCR-dependent responses, which may be critical for naïve B cell unresponsiveness to self-antigens to maintain peripheral tolerance.

At the cellular level, the magnitude and duration of immune responses are regulated by both positive and negative signaling. In B lymphocytes, the relative levels of stimulatory and inhibitory signaling pathways determine the type and amount of antibody produced (1). Mediating such signaling at the B cell surface are mainly immunoreceptor tyrosine-based activation motif (ITAM)-containing stimulatory receptors such as the B cell receptor (BCR) and immunoreceptor tyrosine-based inhibitory motif (ITIM)-containing inhibitory receptors such as CD22 and FcγRIIB. Upon engaging cognate antigens, the ITAMs in the cytoplasmic tails of the BCR complex invariant immunoglobulin (Ig)α and Igβ chains are phosphorylated by SFKs. The dual tyrosines in each ITAM serve as docking sites for the tandem SH2 domains in the spleen tyrosine kinase (Syk). Membrane-docked Syk transmits and amplifies the stimulatory signaling from the BCR to downstream signaling pathways via its tyrosine kinase activity. Conversely, when phosphorylated by Src-family kinases (SFKs), ITIM receptors recruit SH2 domain-containing protein (SHPs) and lipid phosphatases (SHIPs) that directly dephosphorylate various signaling molecules (1).

BCR stimulation-induced positive and negative signals are both dependent upon SFKs. Mature B cells express multiple SFKs including Lyn, Blk, Fyn, Hck, and Fgr, with Lyn being the dominantly expressed form (2). In response to BCR stimulation, Lyn phosphorylates the ITAMs of the BCR Ig domain and the YxxM motif of CD19, allowing for the recruitment of Syk and the docking of PI3K, respectively (3, 4). However, Lyn also phosphorylates inhibitory receptors such as CD22 and FcγRIIB to recruit SHP and SHIP family members, respectively, which exert negative regulatory signals (5, 6). Lyn-deficient B cells are hyperproliferative and exhibit increased Erk phosphorylation and increased calcium in response to BCR stimulation, which reveals that Lyn plays a nonredundant role in activating downstream ITIMs and functions as a net negative regulator of BCR signaling (7).

Since membrane-tethered SFKs can regulate both positive and negative signaling functions downstream of the BCR, the relative activities of SFKs must be well-controlled. In resting cells, SFKs exist in a dynamic steady state between active and inactive conformations. The transitions between the two states are regulated by the phosphorylation of two tyrosine residues, one in the C-terminal tail and the other in the activation loop of the catalytic domain of SFKs. The activation loop phosphorylation, which is positively associated with kinase activity, occurs through transautophosphorylation by an SFK in the active conformation. Phosphorylation at the C-terminal tail tyrosine results in stabilization of a closed, inactive conformation driven by the SH2 and SH3 domains of the SFKs and results in impairment of kinase activity (8). The C-terminal tail tyrosine is phosphorylated predominantly by the C-terminal Src kinase (Csk) and is dephosphorylated by receptor protein tyrosine phosphatases (RPTPs), including CD45 and CD148 in B cells (9, 10). Through the study of mice expressing distinct amounts of these RPTPs, it has been shown that titrating the expression of these RPTPs directly influences the amount of phosphorylation of the C-terminal tail of the B cell SFKs with consequent effects on the activities of BCR signaling as well as B cell development and function (11). Although the expression levels of RPTPs do not change upon BCR stimulation, it is possible that changes in the localization of the RPTPs may occur during B cell interactions with antigen-expressing or antigen-presenting cells, similar to what is observed during kinetic segregation in T cells (12, 13).

The regulation of Csk function is less well understood. Several adaptor molecules including PAG-85, Dok-1 and -2, and others, when phosphorylated by SFKs, have been thought to be part of a negative feedback loop to recruit cytoplasmic Csk to the membrane where SFKs are active (14, 15). However, the specific role of some of these proteins, i.e., PAG-85, has been difficult to validate (16). As Csk regulates SFK activities in both basal and inducible conditions, genetic perturbation of the Csk gene in the hematopoietic lineage results in abnormal development of lymphocytes and thus is unable to fully address the Csk function in normal lymphocytes (17). To overcome this challenge, we have taken advantage of a chemical-genetic system to study the impact of Csk inhibition in B cells. We previously described a transgenic mouse, deficient in the wild-type Csk, that expresses a PP1-analog-sensitive mutant of Csk (Csk^AS^) Bac-transgene (18). The Csk^AS^ mice developed normal lymphoid organs. The Csk^AS^ mutant can be inhibited by a 3-iodobenzyl analog of the kinase inhibitor PP1 (3IB-PP1), which does not inhibit wild-type Csk or other kinases. Our previous studies have shown that 3IB-PP1 inhibition induced SFK activation in Csk^AS^ T cells and weakly induced downstream tyrosine phosphorylation events that partially mimic T cell antigen receptor (TCR) stimulation. However, inhibition of Csk^AS^ by 3IB-PP1 greatly synergized with weak TCR-specific antigenic pMHC or anti-CD3 stimuli (19, 20). It is not clear whether SFK activation induces similar phenotypes in other lymphocytes, although some functional studies in macrophages showed that Csk^AS^ inhibition induced rapid activation of ubiquitin-mediated degradation of SFKs (21). Here, we examined the signaling consequences of Csk inhibition and its impact on SFK activation in B cells. Surprisingly, Csk^AS^ inhibition alone induced marked SFK activation and some downstream phosphorylation, but in contrast to T cells, Csk^AS^ inhibition markedly inhibited simultaneous BCR-mediated cytoplasmic free calcium ([Ca^2+^]_i_) increases and Erk activation. Our studies here reveal that the inhibitory effect of Csk^AS^ inhibition in B cells was caused by hyperactivation of inhibitory receptors and the PIP3 (phosphatidylinositol 3,4,5-triphosphate) lipid phosphatase SHIP1, leading to suppressed PI3K signaling in B cells. We were able to rescue [Ca^2+^]_i_ increases and Erk activation by activating CD19 costimulatory signaling or by deleting SHIP-1. Our findings reveal that Csk promotes BCR signaling by augmenting BCR-mediated PIP3 levels in mature B cells. These features suggest balancing PIP3 levels may help prevent random cell activation resulting from SFK fluctuations in mature B cells. Such control of PIP3 levels via Csk and SFK activities may contribute to the prevention of naïve B cell responses to self-antigen and for establishing self-tolerance.

## Results

### Normal B Cell Development in Csk^AS^ Mice.

To confirm that the introduction of the Csk^AS^ construct and concurrent deletion of wild-type Csk did not alter normal B cell development or signaling in the absence of 3IB-PP1, we first examined the B cell compartment in Csk^AS^ mice. Csk^AS^ mice displayed similar numbers of splenic B cells compared to wild-type mice (*SI Appendix*, Fig. S1*A*). The frequencies of B cells in the marginal zone, transitional, and follicular compartments in Csk^AS^ mice were comparable to those in wild-type mice (*SI Appendix*, Fig. S1*B*). Csk^AS^ follicular B cells expressed amounts of surface BCR receptor IgM, MHC class II, CD86, CD5, and CD23 that were comparable to normal follicular B cells (*SI Appendix*, Fig. S1*C*).

### Inhibition of Csk^AS^ Induces Rapid SFK Activation and Proximal BCR Signals in B Cells.

**Fig. 1.**
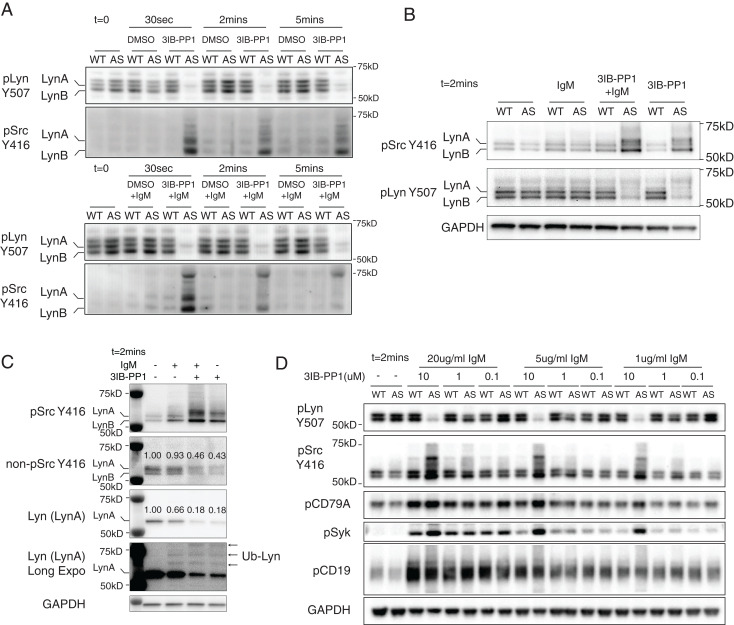
Csk^AS^ inhibition leads to rapid activation of the Src-family tyrosine kinases (SFKs). Total B cells were isolated from wild-type (WT) and Csk^AS^ (AS) lymph nodes and stimulated at 37 °C in vitro. (*A*–*C*) B cells were stimulated with 10 μM 3IB-PP1 and/or 20 μg/mL F(ab′)_2_ anti-μ (IgM). (*A* and *B*) The inactive and active forms of the SFKs (pLyn Y507 and pSFK Y416, respectively) were assessed by immunoblots. The p56 isoform LynA and p53 isoform LynB were marked according to the molecular weight. The pLyn Y507 antibody we used in the blots also reacted with the phosphorylated inhibitory tyrosine sites in other SFKs as well. (*C*) Selected molecular targets were assessed by immunoblot. Of particular note, we used a newly available reagent that detects the nonphosphorylated form of Y416. On non-pSrc Y416 and Lyn (LynA) blots, the relative intensities of each band were measured and marked above each band. On the long-exposure blot of Lyn [Lyn (LynA) Long Expo], mono- and polyubiquitinated LynA bands are indicated with arrows as Ub-Lyn. (*D*) B cells were stimulated with indicated reagents for 2 min. Phosphorylation of the indicated molecular targets was assessed by immunoblots. Data in this figure are representative of two or more independent experiments.

Consistent with our previous findings in T cells and macrophages, acute Csk^AS^ inhibition induced SFK activation in B cells (18–22). As early as 30 s after adding 3IB-PP1 to Csk^AS^ B cells we observed a rapid decrease in phosphorylation of the inhibitory-tail tyrosines on SFKs (as detected by an antibody reactive with phospho-Y507 of Lyn) and strong increases in SFKs activation-loop tyrosine phosphorylation (based on an antibody reactive with phospho-Y416 of Src) (Fig. 1 *A* and *B*). The 3IB-PP1 treatment had no effect on SFK phosphorylation in wild-type B cells, demonstrating the specificity of the analog compound for the Csk^AS^ allele (Fig. 1 *A* and *B*).

Activated Lyn, as assessed by its activation loop phosphorylation, is rapidly ubiquitinated and degraded (23). In Csk^AS^ macrophages, it has been shown that Csk^AS^ inhibition led to rapid ubiquitination of LynA (the long isoform of Lyn) by c-Cbl (21, 24). In B cells, we have observed similar ubiquitination and degradation of LynA following Csk^AS^ inhibition (Fig. 1*C*). As shown on the Lyn (long-exposure) blots, the intensity of the p56 LynA band decreased, and higher molecular bands were detected following 2 min of 3IB-PP1 treatment. Estimated by the decrease of p56 band from the Lyn blot, ∼80% of LynA become activated (as detected with a phospho-Src Y416 antibody) following 2 min of 3IB-PP1 treatment. On the other hand, using an antibody specific to the nonphosphorylated Y416 site (nonpY416) of Src, ∼50 to 60% of nonpY416 Src became phosphorylated following 3IB-PP1 treatment. These results suggest that Csk^AS^ inhibition activates a large proportion of SFKs in B cells.

When cells were treated with 3IB-PP1 in the presence of BCR stimuli [F(ab′)_2_ anti-μ], concomitant Csk^AS^ inhibition induced combinatorial effects with BCR stimulation, inducing robust BCR Ig alpha chain (CD79A) and Syk phosphorylation in a dose-dependent manner (Fig. 1*D*). With weaker BCR stimulation (1 to 5 μg/mL of anti-μ), addition of 10 μM 3IB-PP1 led to strong Syk phosphorylation, similar to the high-dose (20 μg/mL) BCR stimulation. In contrast, 3IB-PP1 treatment did not appear to have a combinatorial effect with BCR stimulation on the phosphorylation of the costimulatory receptor CD19 (Fig. 1*D*).

### Csk^AS^ Inhibition Impairs BCR-Mediated Cytoplasmic Free Calcium Increase and Erk Phosphorylation.

**Fig. 2.**
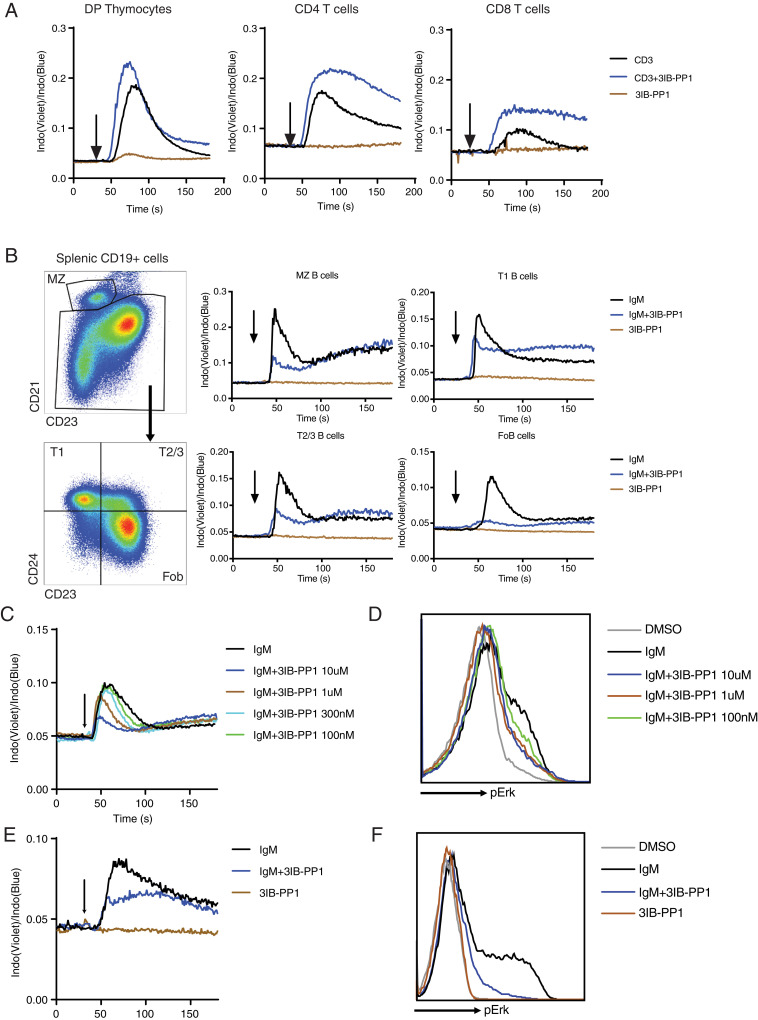
Csk^AS^ inhibition results in impaired BCR-mediated [Ca^2+^]_i_ and Erk activation in B cells. (*A* and *B*) (*A*) CD4+CD8+ double-positive thymocytes (DP Thymocytes), CD4, and CD8 T cells from Csk^AS^ mice were loaded with Indo dye and stimulated with 1 μg/mL anti-CD3 (CD3) and 10 μM 3IB-PP1. (*B*) B cells from Csk^AS^ mice were loaded with Indo dye and stimulated with 20 μg/mL F(ab′)_2_ anti-μ (IgM) and 10 μM 3IB-PP1. Changes in cytoplasmic free calcium ([Ca^2+^]_i_) were assessed by flow cytometry. Arrow indicates addition of reagents. (*C*–*F*) B cells (*C* and *D*) or BAL17-Csk^AS^ cells (*E* and *F*) were stimulated with F(ab′)_2_ anti-μ (IgM) and/or 3IB-PP1. Changes in [Ca^2+^]_i_ and phosphorylation of Erk after 2 min of stimulation were assessed by flow cytometry. The data in this figure are representative of at least two independent experiments.

Ligand-mediated activation of both proximal TCR and BCR pathways typically induces rapid [Ca^2+^]_i_ and phospho-Erk increases, which have critical roles regulating several key transcriptional factor activities in T and B lymphocytes that influence survival, proliferation, and differentiation (25). Consistent with our previous findings (18–20), Csk^AS^ inhibition alone did not increase [Ca^2+^]_I_ in T cells but synergized with anti-CD3 antibodies to induce larger [Ca^2+^]_i_ increases in immature double-positive (CD4+CD8+) thymocytes and mature CD4 and CD8 T cells (Fig. 2*A*). In contrast, in Csk^AS^ B cells, acute Csk^AS^ inhibition led to markedly attenuated BCR-mediated [Ca^2+^]_i_ increases. In marginal zone and transitional B cells, Csk inhibition suppressed the peak [Ca^2+^]_i_ after BCR stimulation. In follicular B cells, 10 μM 3IB-PP1 led to nearly complete inhibition of the BCR-mediated [Ca^2+^]_i_ increase (Fig. 2*B*).

In follicular B cells, titrating the amount of added 3IB-PP1 revealed that acute Csk^AS^ inhibition impairs BCR-induced [Ca^2+^]_i_ increases and Erk phosphorylation in a dose-dependent manner (Fig. 2 *C* and *D*). To further study the inhibitory effect of Csk^AS^ inhibition in B cells, we introduced the Csk^AS^ allele and removed the wild-type Csk allele in a mouse B cell lymphoma cell line, BAL17 (BALENLM 17), which expresses both surface IgM and IgD. Similar to primary B cells, BCR-stimulation on BAL17-Csk^AS^ cells led to large increases in [Ca^2+^]_i_ and phospho-Erk, while addition of 10 μM 3IB-PP1 impaired BCR-mediated [Ca^2+^]_i_ and phospho-Erk increases (Fig. 2 *E* and *F*). These results revealed an unexpected inhibitory role of acute Csk^AS^ inhibition in B cells. These inhibitory effects observed with Csk^AS^ in B cells contrast with the effects of Csk^AS^ inhibition on TCR stimulation in T cells, suggesting some fundamental differences of the proximal BCR and TCR signaling pathways (19, 20).

### Csk^AS^ Inhibition Impairs Cytochalasin-Mediated Cytoplasmic Free Calcium Increase in B Cells.

In the absence of BCR stimulation, it has been reported that disrupting F-actin using cytochalasin D (CytoD) increases BCR diffusion and induces [Ca^2+^]_i_ increases in B cells (26). We also found that CytoD treatment alone induced an increase in [Ca^2+^]_i_ in both immature T1 and, more impressively, in mature follicular B cells, but this was also attenuated by concurrent Csk^AS^ inhibition (*SI Appendix*, Fig. S2 *A*–*C*). Moreover, we observed that although concurrent CytoD treatment led to an increase in BCR-mediated [Ca^2+^]_i_ increase in follicular B cells, these responses (particularly the peak responses) were still impaired by Csk^AS^ inhibition (*SI Appendix*, Fig. S2*C*). Our observations support the previously published studies that cytoskeletal perturbations release the diffusion barriers of BCR and CD19, which can lead to augmentation of BCR signaling. However, we found that Csk^AS^ inhibition still exerts an inhibitory effect independently of actin cytoskeletal effects downstream of BCR and CD19 (27). Thus, Csk^AS^ inhibition impairs CytoD initiated/augmented [Ca^2+^]_i_ in both T1 and follicular B cells.

### Csk^AS^ Inhibition Initiates Negative Regulatory Pathways and Dampens the Amount of PIP3 in B Cells.

Hyperphosphorylation of Igα and Syk induced by Csk^AS^ inhibition alone or with low-dose BCR stimulation is not consistent with the impaired [Ca^2+^]_i_ increases and Erk phosphorylation observed in Csk^AS^ B cells in response to the same treatment conditions. To better understand mechanisms that account for this incongruity, we examined the impact of Csk^AS^ inhibition on the PI3K pathway, which is critical for BCR-mediated [Ca^2+^]_i_ increases. The PI3K pathway is important for regulating the activation, survival, and proliferation of lymphocytes (28). In both T and B cells, PI3K p110α and p110δ are primarily responsible for PIP3 increases, which provides membrane docking sites for various PH domain-containing proteins (29). Specifically, PIP3 facilitates both the Btk kinase and phospholipase C gamma 2 (Plcγ2) positioning at the plasma membrane via their PH domains in B cells (28). PIP3 also recruits Akt via its PH domain to the plasma membrane, where Akt is phosphorylated on threonine 308 by PDK-1 (30). The Thr308 phosphorylation of Akt has been used as an indirect indicator of PIP3 level in cells.

**Fig. 3.**
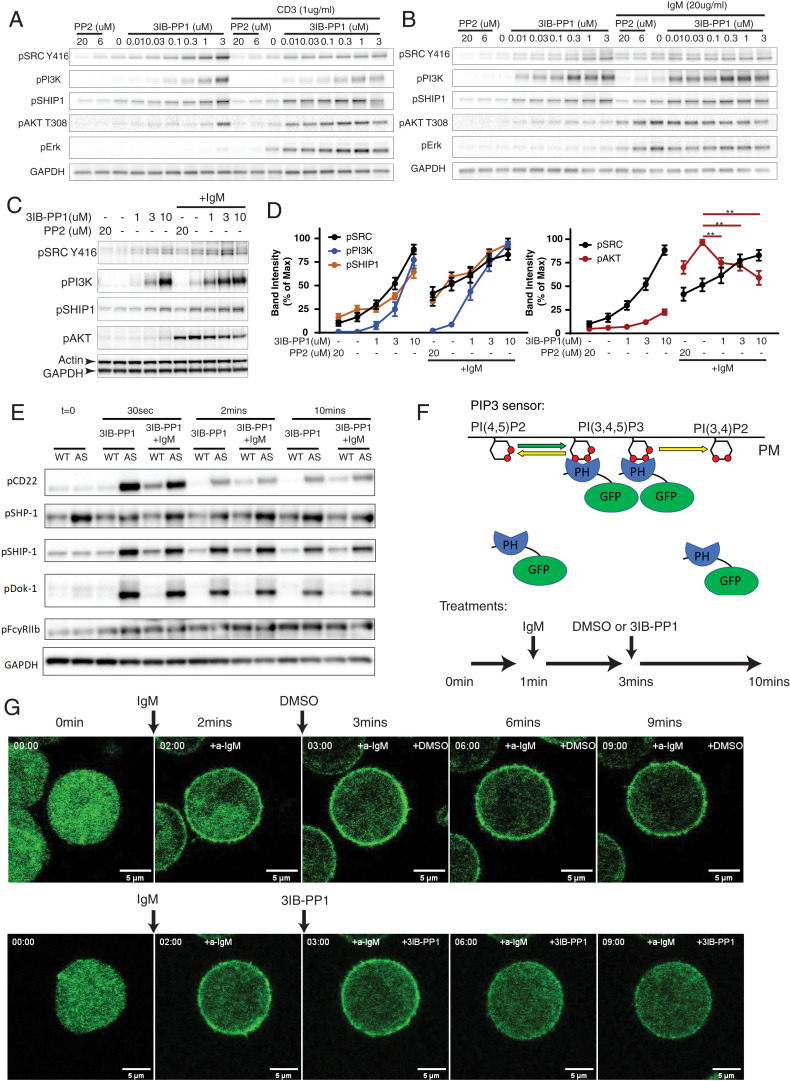
Csk^AS^ inhibition activates ITIM receptors and compromises AKT activation in B cells. (*A*–*B*) Double-positive thymocytes (*A*) and B cells (*B*) isolated from Csk^AS^ mice were stimulated with indicated reagents for 2 min. (*C* and *D*) BAL17-Csk^AS^ cells were stimulated with 10 μg/mL F(ab′)_2_ anti-μ (IgM) and indicated reagents for 2 min. (*C*) Phosphorylation of indicated molecular targets was assessed by immunoblots. (*D*) The band intensities in *C* were quantified and normalized to the band with highest intensity of each molecular target. **P < 0.01 (one-way ANOVA test with Dunnett’s multiple comparisons test, *n* = 8, error bars, SEM). (*E*) B cells from wild-type (WT) and Csk^AS^ (AS) mice were stimulated similarly as in Fig. 1*A*. Phosphorylation of selected ITIM receptors and associated phosphatases were assessed by immunoblots. (*F* and *G*) BAL17-Csk^AS^ cells were transfected with a PIP3 GFP biosensor. The cells were stimulated with 20 μg/mL F(ab′)_2_ anti-μ (IgM) and 10 μM 3IB-PP1 or DMSO for the indicated time intervals (*F*). The translocation of the PIP3 sensor was imaged by confocal microscopy continuously for 10 min. Selected key frames are shown (*G*). Full movie images are presented in Movies S1 and S2. All data are representative of at least two independent experiments.

Both TCR and BCR stimulation activates PI3K by phosphorylating PI3K p85/p55 regulatory subunits in an SFK-dependent manner (28). In Csk^AS^ thymocytes and B cells, antigen receptor clustering using either CD3 and IgM antibodies led to phosphorylation of PI3K p85/p55 subunits and phospho-Akt (Fig. 3 *A* and *B*). We treated cells with 3IB-PP1 or the SFK inhibitor PP2 to positively or negatively regulate SFK activities, respectively, which was monitored by phosphorylation of the SFK activation loop tyrosine using a Src pY416-specific antibody. We found that induction of phospho-PI3K correlated with SFK activity in both thymocytes and B cells. In thymocytes, stronger phospho-PI3K also correlated with increased phospho-Akt (Fig. 3*A*). However, in B cells Csk^AS^ inhibition increased SFK activity induced by BCR stimulation, but impaired phosphorylation of Akt despite the hyperphosphorylation of PI3K (Fig. 3*B*). Similarly, in BAL17-Csk^AS^ cells that were stimulated via their BCR, 3IB-PP1 increased SFK activation loop phosphorylation but impaired the phosphorylation of Akt (Fig. 3 *C* and *D*).

The mouse genome encodes multiple negative regulators of PIP3. The SH2-containing inositol polyphosphate 5-phosphatase (SHIP1) is one of the most highly expressed phosphatases that targets PIP3 in B cells (31). SHIP1 opposes the actions of PI3K by catalyzing the dephosphorylation of PI(3,4,5)P3 into PI(3,4)P2. In both thymocytes and B cells, Csk^AS^ inhibition induced strong phosphorylation of the SHIP1 C-terminus tyrosine (Y1020), which is critical for SHIP1 binding to Dok-1 near the plasma membrane (31) (Fig. 3 *A*, *B*, and *E*). However, the similarly up-regulated phospho-SHIP1 does not explain the different roles of Csk^AS^ inhibition between T cells and B cells. In contrast to T cells, B cells express the transmembrane ITIM receptors FcγRIIB and CD22, as well as the cytosolic ITIM containing adaptor Dok-1, which all have been reported to contribute to recruiting SHIP1 to the plasma membrane (32, 33). Following Csk^AS^ inhibition, the ITIM tyrosines of CD22 and Dok-1 were rapidly phosphorylated in Csk^AS^ B cells, whereas the ITIM tyrosine of FcγRIIB was less so (Fig. 3*E*). The hyperphosphorylated SHIP1 and its binding partners CD22 and Dok-1, in particular, may explain the inhibitory effect of PIP3 levels in Csk^AS^-inhibited B cells.

Unlike SHIP1, the phosphorylation of SH2 domain-containing tyrosin phosphatase SHP-1 was only weakly elevated following Csk^AS^ inhibition in B cells, which suggests that SHP-1 is less involved in the Csk^AS^ inhibition pathway (Fig. 3*E*). In Csk^AS^ B cells, we have repeatedly observed a mildly higher degree of SHP-1 phosphorylation (pSHP-1) level in the basal state compared with wild-type B cells. The reason for the elevated pSHP-1 is not fully understood. It may represent an adaptation to the somewhat reduced kinase activity of the Csk^AS^ allele compared with the wild-type Csk allele (18). It should be noted that the pSHP-1 blots in Fig. 3*E* are relatively long-exposure blots, and the overall pSHP-1 signal is very weak in Csk^AS^ B cells with or without Csk^AS^ inhibition.

CD22 is one of the best characterized ITIM-containing receptors in B cells. Phosphorylation of the ITIMs of CD22 serve as docking sites for both tyrosine phosphatase SHP-1 and lipid phosphatase SHIP1 (33, 34). CD22 restrains both BCR and cytoskeleton disruption-induced signaling in B cells (35). To test whether the inhibitory effect of 3IB-PP1 treatment is dependent on CD22, we crossed the Csk^AS^ mice with CD22-deficient (CD22^−/−^) mice (36). 3IB-PP1 treatment still inhibited BCR-mediated [Ca^2+^]_i_ and phospho-Erk increases in CD22-deficient Csk^AS^ B cells to a similar extent as in CD22 sufficient cells (*SI Appendix*, Fig. S3 *A* and *B*). These findings in the CD22^−/−^Csk^AS^ B cells suggested the negative regulatory effects of Csk^AS^ inhibition in B cells are not dependent only on CD22.

To further test whether the suppressed phospho-Akt in Csk^AS^-inhibited B cells represented a decrease in PIP3 levels at the plasma membrane, we expressed a fluorescent sensor of PIP3 in the BAL17-Csk^AS^ cells. The PIP3 sensor consists of the PIP3 binding PH domain of Akt fused to a green fluorescent protein (GFP) (37). The sensor translocates between the plasma membrane and cytosol in cells with high and low PIP3 amounts, respectively (Fig. 3*F*). Shortly after the PIP3 sensor-expressing BAL17-Csk^AS^ cells were treated with anti-μ, the PIP3 sensor was rapidly enriched at the plasma membrane, indicative of cells producing a high amount of PIP3 in the plasma membrane. The membrane-bound PIP3 sensor reached its peak level approximately 2 min after the anti-μ treatment. Addition of 3IB-PP1 at this time point resulted in translocation of PIP3 sensors back to the cytosol, whereas the PIP3 sensor remained enriched at the plasma membrane in BCR-stimulated cells treated with dimethyl sulfoxide (DMSO) (Fig. 3*G* and Movies S1 and S2). The dynamics of PIP3 levels on the cell plasma membrane correlates with the [Ca^2+^]_i_ in similarly stimulated cells in a parallel experiment (*SI Appendix*, Fig. S4). These results suggest that Csk^AS^ inhibition, which leads to increased SFK activity, impairs BCR-mediated PIP3 accumulation in B cells. This could contribute to the overall negative regulatory effect of Csk^AS^ inhibition on [Ca^2+^]_i_ and phospho-Erk increases in B cells.

### The Negative Regulatory Effects of Csk^AS^ Inhibition Are Not Solely Dependent on Lyn.

**Fig. 4.**
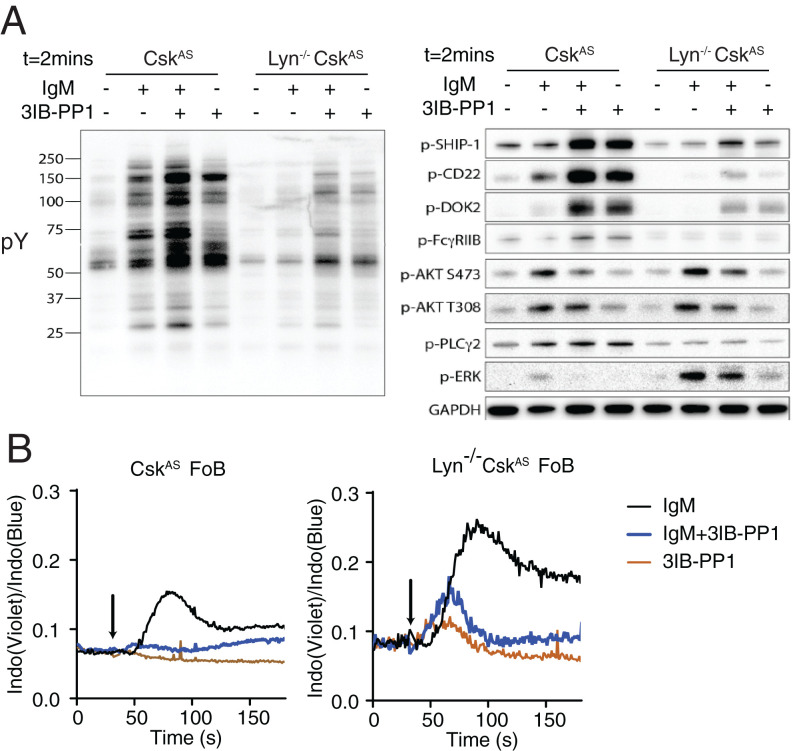
The inhibitory effects of Csk^AS^ inhibition are not dependent on Lyn. B cells isolated from Csk^AS^ and Lyn^−/−^ Csk^AS^ mice were stimulated with 20 μg/mL F(ab′)_2_ anti-μ (IgM) and 10 μM 3IB-PP1 for 2 min. (*A*) Total phosphotyrosine proteins (pY) and phosphorylation of selected molecular targets were assessed by immunoblot. (*B*) Follicular B cells (FoB) were loaded with Indo dye and stimulated with 20 μg/mL F(ab′)_2_ anti-μ (IgM) and 10 μM 3IB-PP1. [Ca^2+^]_i_ was assessed by flow cytometry. Arrow indicates addition of reagents. Data in this figure are representative of at least two independent experiments.

Lyn is the most predominant SFK expressed in B cells and phosphorylates both ITAM- and ITIM-containing receptors with a net negative regulatory role in BCR signaling (38). To examine whether the negative regulatory effects of Csk^AS^ inhibition in B cells are dependent on the activity of Lyn kinase, Csk^AS^ mice were crossed to the Lyn-deficient (Lyn^−/−^) background. We found that deletion of Lyn resulted in global decrease of BCR-mediated tyrosine phosphorylation but also an increase of [Ca^2+^]_i_ in B cells, which was consistent with previous studies (Fig. 4 *A* and *B*) (7). Surprisingly, in Lyn^−/−^ Csk^AS^ cells, 3IB-PP1 treatment failed to up-regulate the phosphorylation of FcγRIIB but still elevated the phosphorylation of CD22, Dok2, and SHIP1 and suppressed phospho-Akt upon BCR stimulation (Fig. 4*A*). Increased phospho-SHIP1 and decreased phospho-Akt still correlated with impaired [Ca^2+^]_i_ and phospho-Erk increases in 3IB-PP1–treated Lyn^−/−^ Csk^AS^ cells (Fig. 4 *A* and *B*). To determine whether the tyrosine phosphorylation induced by 3IB-PP1 occurs via other SFKs in the Lyn^−/−^ B cells, both wild-type and Lyn^−/−^ Csk^AS^ B cells were pretreated with SFK inhibitor PP2 for 15 min and then stimulated with anti-IgM antibodies. The tyrosine phosphorylation induced by 3IB-PP1 in Lyn^−/−^ B cells was largely eliminated, suggesting the role of 3IB-PP1 is mediated via SFK activation (*SI Appendix*, Fig. S5). These findings suggested the inhibitory effects of Csk^AS^ inhibition in B cells are not dependent only on the Lyn–FcγRIIB–SHIP1 axis. Other SFKs, such as Blk and Fyn, may play compensatory roles in phosphorylating CD22, Dok2, and SHIP1 and exert negative regulation of PIP3 amounts in B cells.

### CD19 Costimulation Overcomes the Negative Regulatory Effects of Csk^AS^ Inhibition.

**Fig. 5.**
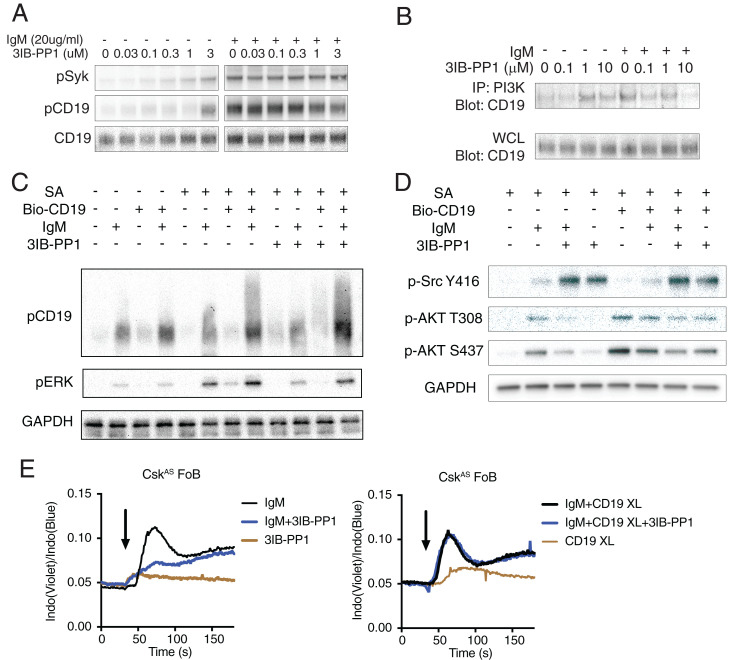
CD19 costimulation restores BCR-mediated [Ca^2+^]_i_ in B cells. (*A*) B cells from Csk^AS^ mice were stimulated with indicated reagents for 2 min. (*B*) PI3K p85 and p55 subunits were immunoprecipitated by an antibody mixture of anti-p85 and anti-p55 antibodies. CD19 immunoprecipitated and in the whole-cell lysates (WCL) were detected by immunoblots. (*C*–*D*) B cells from Csk^AS^ mice were incubated with biotin-conjugated anti-CD19 antibody (Bio-CD19) at 37 °C for 15 min. Cells were subsequently stimulated with 20 μg/mL F(ab′)_2_ anti-μ (IgM), 10 μM 3IB-PP1, and streptavidin cross-linker (SA) for 2 min. Selected molecular targets were assessed by immunoblot. (*E*) Cells were loaded with Indo dye and stimulated with 20 μg/mL F(ab′)_2_ anti-μ (IgM) and 10 μM 3IB-PP1 and [Ca^2+^]_i_ in the follicular B cell compartment was assessed by flow cytometry. Arrow indicates addition of the reagents. Data in this figure are representative of at least two independent experiments.

BCR signaling can be amplified through the costimulatory receptor CD19. CD19 is a transmembrane glycoprotein which forms a unique complex with CD21 and CD81 on the B cell surface (3). Two YxxM motifs in the cytoplasmic tail of CD19 can be tyrosine-phosphorylated by SFKs and bind the PI3K p85 SH2 domains with high affinity (39, 40). Although the membrane-associated protein BCAP may also recruit p85 during early B cell development, BCAP is not essential for PI3K activity in mature B cells (41). In Csk^AS^ B cells, a high concentration of 3IB-PP1 treatment can induce moderate CD19 phosphorylation. However, in contrast with Igα of the BCR, concomitant treatment of 3IB-PP1 with BCR stimulation did not have an additive effect on CD19 phosphorylation (Fig. 5*A*). Moreover, a high dose of 3IB-PP1 impaired BCR-mediated interaction between CD19 and PI3K (Fig. 5*B*). To examine whether the uncoupling of the CD19 signals causes the blockade of downstream calcium and Erk signal, we cross-linked CD19 on B cells. Cross-linking CD19 restored the BCR-mediated Erk phosphorylation in the presence of 3IB-PP1 (Fig. 5*C*). Cross-linking CD19 in the presence of 3IB-PP1 treatment elevated Akt phosphorylation to a similar extent as with BCR stimulation in Csk^AS^ B cells, which suggested CD19 cross-linking can induce an increase of PIP3 on the plasma membrane, even in the presence of 3IB-PP1 (Fig. 5*D*). With the elevated PIP3 amount, CD19 cross-linking diminished the negative regulatory effect of Csk^AS^ inhibition on intracellular calcium mobilization. BCR stimulation induced robust [Ca^2+^]_i_ increase in Csk^AS^ B cells treated with 3IB-PP1 in which CD19 was also cross-linked (Fig. 5*E*), demonstrating that CD19 cross-linking can reverse the negative regulatory effects of Csk^AS^ inhibition, likely by increasing additional PIP3 in B cells. These data support our hypothesis that the negative regulatory effects of Csk^AS^ inhibition in B cells are caused by suppression of PIP3 levels in B cells.

### SHIP1 Partially Mediates the Inhibitory Effects of Csk^AS^ Inhibition in B Cells.

**Fig. 6.**
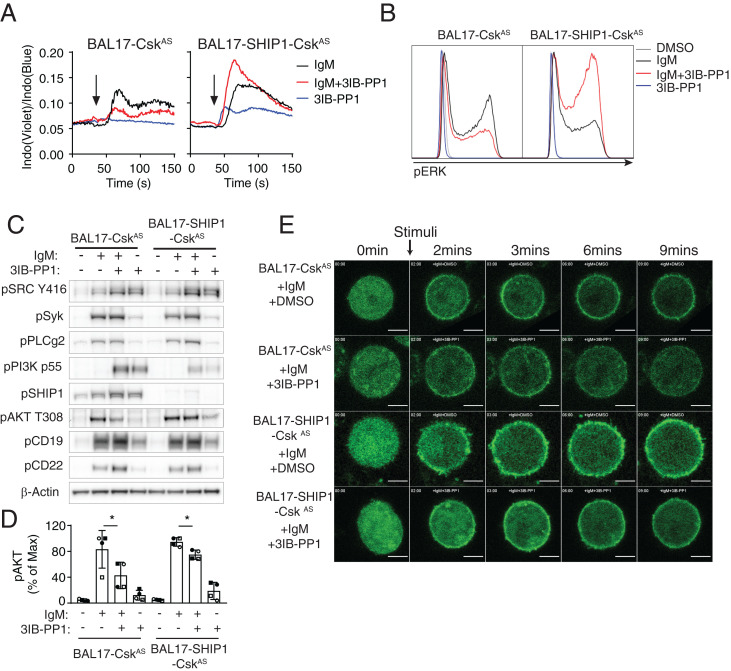
The inhibitory effect of Csk^AS^ inhibition is dependent on SHIP1. BAL17-Csk^AS^ and BAL17-SHIP1-Csk^AS^ cells were stimulated with 20 μg/mL F(ab′)_2_ anti-μ (IgM) and 10 μM 3IB-PP1. Changes in [Ca^2+^]_i_ (*A*) and phosphorylation of Erk (*B*) after 2-min stimulation was assessed by flow cytometry. (C) Phosphorylation of selected molecular targets after 2-min stimulation was assessed by immunoblot. Data in this figure are representative of at least two independent experiments. (*D*) The band intensities of pAKT T308 from four independent experiments similar to *C* were quantified and normalized to the most intense band. Samples from the same experiment were represented by the same symbol (open circle, closed circle, open square, or closed square). *P < 0.05 (paired *t* test, *n* = 4, error bars, SEM). (*E*) BAL17-Csk^AS^ and BAL17-SHIP1-Csk^AS^ cells expressing PIP3 GFP biosensors were stimulated with 20 μg/mL F(ab′)_2_ anti-μ (IgM) and 10 μM 3IB-PP1 or DMSO for the indicated time intervals. The translocation of the PIP3 sensor was imaged by confocal microscopy continuously for 10 min. Several key frames are shown. Full movies are presented in Movies S3–S6. Images are representative of at least ten regions of interest from two independent experiments. (Scale bars: 5 μm.)

Among several negative signaling molecules activated by Csk^AS^ inhibition, SHIP1 is one of the main negative regulators of PIP3 level in B cells. We hypothesized that Csk^AS^ inhibition led to activation of SHIP1, which mediated the negative regulatory effects in B cells. To examine whether SHIP1 is the bona fide downstream effector mediating the negative regulatory effects of Csk^AS^ inhibition in B cells, we generated SHIP1-deficient BAL17-Csk^AS^ cells (BAL17-SHIP1-Csk^AS^). Csk^AS^ inhibition in BAL17-Csk^AS^ cells impaired BCR-mediated [Ca^2+^]_i_ increase and pErk increases as well as pAKT increases (Fig. 6 *A* and *D*). On the other hand, Csk^AS^ inhibition in BAL17-SHIP1-Csk^AS^ cells boosted BCR-mediated increases of [Ca^2+^]_i_ and phospho-Erk (Fig. 6 *A* and *B*). It was notable that Csk^AS^ inhibition also decreased phospho-Akt in BAL17-SHIP1-Csk^AS^ cells, but to a lesser extent compared with in BAL17-Csk^AS^ cells (Fig. 6 *C* and *D*). To test whether the phosphorylation of Akt in these cell lines represents PIP3 level on the plasma membrane, the GFP-tagged PIP3 biosensor was expressed in both cell lines, which were subsequently stimulated similarly as in Fig. 6*A*. In the presence of BCR stimulation, 3IB-PP1 treatment led to rapid translocation of the biosensor from the plasma membrane to the cytosol in BAL17-Csk^AS^ cells but not in BAL17-SHIP1-Csk^AS^ cells (Fig. 6*E* and Movies S3–S6). These results strongly suggest that SHIP1 mediates the inhibitory effects of Csk^AS^ inhibition on BCR signaling by regulating PIP3 levels. The finding that 3IB-PP1 treatment was still able to partially decrease phospho-Akt in the absence of SHIP1 suggests that there are other negative regulators downstream of Csk regulating PIP3 levels in B cells. Mature B cells express SHIP2 and other inositol polyphosphate phosphatases which may play redundant roles in dephosphorylating PIP3 and dampening BCR signaling.

## Discussion

Here, we have studied the effects of rapid increases in SFK activity by inhibition of Csk kinase activity in B cells using BAL17 cells and mice expressing a mutant Csk allele, Csk^AS^, that is sensitive to a small molecule kinase inhibitor 3IB-PP1. Inhibition of Csk^AS^ in the presence of BCR stimuli causes rapid dephosphorylation of C-terminal tail tyrosines of SFKs and induces phosphorylation of the activating SFK tyrosines in B cells. This leads to the hyperactivation of SFKs, which results in robust phosphorylation of BCR ITAMs and proximal BCR components in the absence of antigen receptor stimulation. However, unlike parallel observations we made in T cells, Csk^AS^ inhibition substantially suppressed the BCR-mediated [Ca^2+^]_i_ and phospho-Erk increases in primary mature B cells and in BAL17 cells. This disconnect between hyperactivation of SFKs and the downstream inhibition of [Ca^2+^]_i_ and phospho-Erk increases appears to be mediated by a negative regulatory circuit targeting the PI3K–PIP3 pathway that was not previously recognized (*SI Appendix*, Fig. S6).

Our findings reveal that Csk^AS^ inhibition and consequent increases in SFK activity result in different signaling outcomes in B cells and T cells. This raises questions regarding how and why BCR and TCR proximal signaling develop different regulatory circuits to buffer changes in SFK activities. One of the main differences between BCR and TCR pathways is the differentially expressed SFKs family members in the two lineages. Lck is the dominant SFK in T cells, while B cells express large amounts of Lyn. Lck and Lyn both activate positive as well as negative regulatory signaling pathways, but genetic ablation of Lck or Lyn results in opposite effects in T and B cells. Lck deficiency results in impaired TCR signaling and a partial blockade of αβ T cell development, demonstrating that Lck acts as a positive regulator during T cell development and in naïve peripheral T cell function (42, 43). Lyn-deficient B cells, on the other hand, are found to be hyperresponsive to BCR stimuli, which suggests Lyn acts predominantly as a net negative regulator in naïve B cells (7). Moreover, mice harboring a constitutively active allele of Lyn (*Lyn^up/up^*) exhibit features of B cell anergy which imitates many aspects that we observed when Csk^AS^ was inhibited in our study (44). The net negative effects of Lyn have been linked to activation of inhibitory receptors such as FcγRIIB and CD22 (38, 45, 46). In our study, Csk^AS^ inhibition leads to global SFK activation in B cells and results in rapid phosphorylation of FcγRIIB and CD22. We initially thought the inhibitory effect of Csk^AS^ inhibition was likely mediated by activation of Lyn. However, in Lyn-deficient B cells, Csk^AS^ inhibition also attenuated BCR ligation-induced [Ca^2+^]_i_ and phospho-Erk increases. Our results suggest other B cell-expressed SFKs, such as Blk and Fyn, can also engage inhibitory pathways in B cells in the absence of Lyn.

Naïve resting B cells express many inhibitory receptors, in contrast to naïve resting T cells. One explanation for this interesting phenomenon is that Syk, the kinase immediately downstream of SFKs in B cells, is less autoinhibited than Zap70, which is expressed in T cells (47). This may be due to increased conformational flexibility of Syk than of Zap70 (48). In T cells, the two SH2 domains of Zap70 are tightly associated with the kinase domain in an autoinhibited conformation (49). Moreover, as a tandem unit, the SH2 domains of Zap70 bind to phospho-ITAMs with high affinity upon TCR engagement (50), whereas in B cells the two SH2 domains of Syk are more loosely aligned (48, 51). The conformational flexibility results in less autoinhibition of Syk and more basal activity. Moreover, unlike Zap70, Syk can substitute for SFKs and phosphorylate ITAMs in B cells when polyvalent antigens or cross-linked antibodies are used for stimulation, whereas Zap70 cannot (52). When overexpressed in T cells, Syk, but not Zap70, can induce strong TCR-induced signals independent of SFKs (53). Thus, it is reasonable to assume that B cells require more control by inhibitory receptors to suppress the higher basal activity of Syk. In our study, we have shown that Csk^AS^ inhibition results in rapid phosphorylation of inhibitory receptors and suppression of the BCR-mediated signals. This could be a part of a BCR proofreading mechanism.

In the immune synapse, segregation of CD22 is observed, accompanied by colocalization of the BCR and CD19 (54). The molecular mechanism of CD19–BCR clustering upon BCR aggregation is not clear, but a recent study of CD19–CD81 complex structure might shed some light on this issue (55). The tetraspanin CD81 is an important binding partner for CD19 and is required for its trafficking to the cell surface (56). On the plasma membrane, CD81 regulates the diffusion of CD19 by immobilizing CD19, which may prevent CD19 engagement with the BCR (27). A key regulator that has been shown to disrupt the CD19–CD81 complex is cholesterol. Molecular dynamics simulations suggest CD81 adopts a closed conformation which binds cholesterol, but not CD19, and an open conformation which prefers CD19 to cholesterol (55). In mature B cells, the BCR translocates into cholesterol-enriched microdomains on the plasma membrane shortly after BCR aggregation (57). These cholesterol-enriched microdomains may facilitate the adoption of CD81 into its closed conformation, thereby releasing CD19 from CD81 and enhancing the clustering of CD19 and BCR. The spatial regulation of costimulatory receptors, such as CD19, and inhibitory receptors in a bona fide B cell immune synapse may be the key to fully activating BCR signals. In our Csk^AS^ model, we cross-linked the stimulatory receptor CD19, which could at least partially overcome the inhibitory effect of Csk^AS^ inhibition.

The different outcomes of Csk^AS^ inhibition in T and B cells seem to be rooted in their differentially regulated PI3K pathways. Although PIP3 is a critical signaling molecule for both TCR and BCR signaling, B cell development relies more heavily on PI3K activity than do T cells. Combined deletion of both p110α and p110δ, the two main PI3K isoforms expressed in both B and T cells, results in a severe block for B cell development at the pre-B cell stage, whereas T cell development is not greatly impacted (29, 58). Given the critical importance of PIP3, B cells have developed more stringent molecular circuits to sense and regulate PIP3 than have T cells. A good example is Btk in B cells, which can undergo a dimerization-mediated activation that sharply increases its sensitivity to PIP3, which is in contrast to Tec and Itk in T cells (59). Hence, this could be a driver in B cells to express more inhibitory molecules in order to provide more regulation of PIP3 levels than in T cells. In lymphocytes, the main negative regulators of PIP3 are phosphoinositide 3-phosphatase PTEN and the SH2 domain containing inositol polyphosphate 5-phosphatase SHIP1 and SHIP2. T cells express PTEN and SHIP1 and B cells express all three phosphatases. Unlike PTEN, whose phosphatase activity and subcellular location are not heavily regulated by TCR or BCR signaling over the short term, SHIP phosphatases are recruited to the plasma membrane and access the lipid substrate PIP3 shortly after antigen receptor stimuli. The N-terminal SH2 domain of SHIP phosphatases binds to phosphorylated ITIMs receptors such as FcγRIIB and CD22 following BCR stimulation of naïve B cells (32, 60). In T cells, although the ITAMs in the CD3 complex can bind SHIP1 in vitro, it is not clear to what extent SHIP1 can be recruited to the CD3 in cells since SHIP1 needs to compete for the same ITAMs with the tandem SH2 domains of Zap70, which bind with very high affinity (61). Therefore, ITIM-containing receptors may not play as important a role in naïve resting T cells in regulating the PI3K pathway. In our study, Csk^AS^ inhibition leads to rapid phosphorylation of multiple ITIMs and SHIP1 in B cells. The activation of this inhibitory feedback loop dampens PIP3 levels in the plasma membrane and prevents downstream signals from propagating in reponse to SFK activation in the absence of transient strong BCR stimulation. The circuitry of mature B cells involving Csk, SFK, and PIP3 may contribute to the biochemical mechanisms of B cell anergy. Indeed, PIP3- and ITIM-dependent signaling have been tightly connected with B cell anergy (62, 63). Inducible deletion of SHIP1 in adoptively transferred mature Ars/A1 B cells reversed the anergic phenotype in a murine model of B cell anergy (62). Overall, our results suggest Csk can act as an upstream regulator of PI3K regulatory pathways in resting naïve B cells but may not be needed to do so in naïve T cells.

In sum, our studies highlight the importance of coordinated functional action of SFKs in the regulation of proximal signaling by the BCR. Compared with other immune cells, the net result of tuning SFK activity by Csk inhibition could vary dramatically due to the different downstream PIP3 signaling circuitry in B versus T cells, for example. The different effects of Csk inhibition in T and B cells could have potential downstream therapeutic considerations. For example, in the setting of B cell lymphoma, delivery of a small-molecule Csk inhibitor could augment a tumor-specific T cell response while inhibiting the growth of transformed B cells by blocking their PI3K pathway.

## Materials and Methods

### Mice.

Mice used in these experiments were 6 to 12 wk of age. Csk^AS^ mice are heterozygous for a BAC transgenic Csk^AS^ allele on a Csk-null background (18). Cd22^−/−^ mice (36) and Lyn^−/−^ mice (7) were used for breeding. Cd22^−/−^Csk^AS^ mice were generated by crossing Csk^AS^ and Cd22^−/−^ mice. Lyn^−/−^Csk^AS^ mice were generated by crossing Csk^AS^ and Lyn^−/−^ mice. All animals were maintained on a C57BL/6 genetic background. All mice were housed in a specific pathogen-free facility at the University of California San Francisco (UCSF) according to the University Animal Care Committee and NIH guidelines.

### Antibodies, Dyes, and Inhibitors.

Antibodies to murine B220, CD19, CD5, CD23, CD21, IgM, CD69, CD11b, CD22, CD24, CD45, CD86, phospho-PLCγ2 (Y759), and phospho-Syk (Y352) were from BD Biosciences. Antibodies against phospho-CD79a(Y182), phospho-Erk (T202/Y204), phospho-Src family (Tyr416), total Lyn, phospho-CD19 (Y531, PI3K-activation motif), GAPDH, phospho-PI3K p85 (Y458)/p55 (Y199), phospho-SHIP1 (Y1020), phospho-Akt (T308), phospho-Akt (S473), phospho-SHP-1 (Y564), nonphosphorylated Src Tyr416 (clone: 7G9), and phospho-Lyn (Tyr507) were from Cell Signaling Technology. Antibodies to phospho-CD22 (Y506, ITIM motif) and phospho-Dok1 (Y362) were from Abcam. Antibody to Phospho-FcgammaRIIb/CD32 (Y292) was from Epitomics. Antibody to phosphotyrosine (clone: 4G10) was from Millipore. Goat anti-mouse F(ab′)2 anti-μ and strepavidin were from Jackson Immunoresearch. 3IB-PP1 (3-Iodobenzyl PP1 Analog, PP1 Analog IV) was from US Biological Life Sciences. Indo-1-AM dye was from Thermo Fisher Scientific.

### Cell Lines and CRISPR/Cas9.

BALENLM 17 (BAL17) cell lines were provided by the Anthony DeFranco laboratory at UCSF. Csk^AS^-expressing BAL17 cells were generated by electroporating Csk^AS^-mCherry bearing pEF6-HisA plasmid. Five days after electroporation, mCherry-expressing cells were enriched by FACS sorting using a BD FACS ARIA. The enriched cells were subsequently electroporated with a pX330 vector bearing a guide RNA (gRNA) targeting endogenous Csk but not the Csk^AS^. Cells were cultured for 2 d and sorted into 96-well flat-bottom plates using a single-cell sorting protocol. BAL17-Csk^AS^ clones were screened and picked for deficiency of endogenous Csk protein but expressing Csk^AS^-mCherry protein. BAL17-SHIP1-Csk^AS^ cells were generated with similar procedures using a pX330 vector bearing a gRNA targeting the SHIP1 gene locus. All cells were maintained in a tissue culture incubator at 37 °C with 5% CO_2_ in culture medium (RPMI supplemented with 10% fetal bovine serum, 2 mM glutamine, 1 mM sodium pyruvate, and 55 nM 2-mercaptoethanol).

### B Cell Isolation and Immunoblotting.

Primary B cells were isolated from spleen and lymph node cell suspensions using MACS B cell isolation kits (130-090-862; Miltenyi Biotec). Isolated B cells were resuspended at 10 × 10^6^ cells/mL for stimulation. BAL17 cells were rinsed with RPMI and resuspended at 5 × 10^6^ cells per mL. All cells used for experiments were rested for 15 min at 37 °C prior to stimulation. Cells were subsequently treated with stimulating reagents and then lysed by the addition of lysis buffer containing a final concentration of 1% Nonidet P-40, NaVO_4_ (2 mM), NaF (10 mM), ethylenediaminetetraacetic acid (EDTA) (5 mM), phenylmethanesulfonyl fluoride (2 mM), aprotinin (10 μg/mL), pepstatin (1 μg/mL), leupeptin (1 μg/mL), and PP2 (25 μM). Lysates were placed on ice and centrifuged at 13,000 × *g* for 10 min at 4 °C. Supernatants were run on 4 to 12% NuPage (Invitrogen) and transferred to polyvinylidene fluoride membranes. Membranes were incubated with blocking buffer (1% bovine serum albumin in Tris-buffered saline, 0.05% Tween 20, pH 7.4) and then probed with primary antibodies overnight at 4 °C. The following day blots were rinsed and incubated with HRP-conjugated secondary antibodies (diluted 1:10,000). Blots were detected using chemiluminescent substrate and a Chemi-Doc (Bio-Rad) imaging system. Quantification was performed using imaging processing software (Adobe Photoshop).

### Calcium Assays.

Cells were loaded with the Indo1-AM (1.5 μM; Invitrogen) for 30 min at 37 °C in RPMI with 10% fetal bovine serum, washed, surface-stained, and kept on ice in RPMI. Cells were warmed to 37 °C for five minutes before stimulation. Changes in Indo1 fluorescence in cells were either recorded using a FlexStation or FACS setup using a BD LSRFortessa. FACS data were exported from FlowJo in CSV format and analyzed with an R script (https://github.com/richard02050411/Calcium-Flux-FACs/blob/master/R-code) Graphs were generated using GraphPad Prism software.

### Phospho-Erk Staining.

Cells were stimulated in RPMI and fixed by adding BD Fixation/Permeabilization solution (1:1 ratio, paraformaldehyde-based fixation reagent, 554714; BD) and incubating for ten minutes at room temperature. Cells were pelleted and rinsed with FACS buffer (PBS supplemented with 2% fetal bovine serum and 2 mM EDTA). Cells were then placed on ice and ice-cold 90% methanol was added to permeabilize the cells for 45 min. Cells were then rinsed three times with FACS buffer and resuspended in staining solution (anti-phospho-Erk 1:100 in FACS buffer). Cells were stained for either 1 h at room temperature or overnight at 4 °C. Cells were rinsed three times and stained with anti-rabbit PE antibody and anti-CD45 AF647 antibody (1:100 in FACS buffer) for 30 min at room temperature. Cells were rinsed twice and analyzed by flow cytometry using a BD Fortessa and quantification performed using FlowJo software. For Csk inhibitor dose–response curves, data were fit using agonist versus response with variable slope in GraphPad Prism.

### Microscopy.

PIP3 sensor-expressing cells were plated on glass surfaces coated with 5 μg/mL Icam1 for 30 min at 37 °C. Cells were imaged with the Nikon A1R laser scanning confocal fluorescence microscopy with NIS-Elements software and a 100× oil dipping objective. The data were processed by ImageJ software.

## Data Availability

All study data are included in the article and/or supporting information.
